# Genetic diversity in vector populations influences the transmission efficiency of an important plant virus

**DOI:** 10.1098/rsbl.2024.0095

**Published:** 2024-05-22

**Authors:** Daniel J. Leybourne, Mark A. Whitehead, Torsten Will

**Affiliations:** ^1^ Department of Evolution, Ecology and Behaviour. Institute of Infection Veterinary and Ecological Science, University of Liverpool, Liverpool L69 7ZB, UK; ^2^ Centre for Genomics Research. Institute of Infection Veterinary and Ecological Science, University of Liverpool, Liverpool L69 7ZB, UK; ^3^ Institute for Resistance Research and Stress Tolerance, Julius Kühn-Institute – Federal Research Centre for Cultivated Plants, Quedlinburg 06484, Germany

**Keywords:** barley yellow dwarf virus, plant virology, vector–virus interactions, aphid, transmission efficiency

## Abstract

The transmission efficiency of aphid-vectored plant viruses can differ between aphid populations. Intra-species diversity (genetic variation, endosymbionts) is a key determinant of aphid phenotype; however, the extent to which intra-species diversity contributes towards variation in virus transmission efficiency is unclear. Here, we use multiple populations of two key aphid species that vector barley yellow dwarf virus (BYDV) strain PAV (BYDV-PAV), the grain aphid (*Sitobion avenae*) and the bird cherry-oat aphid (*Rhopalosiphum padi*), and examine how diversity in vector populations influences virus transmission efficiency. We use Illumina sequencing to characterize genetic and endosymbiont variation in multiple *Si. avenae* and *Rh. padi* populations and conduct BYDV-PAV transmission experiments to identify links between intra-species diversity in the vector and virus transmission efficiency. We observe limited variation in the transmission efficiency of *Si. avenae,* with transmission efficiency consistently low for this species. However, for *Rh. padi,* we observe a range of transmission efficiencies and show that BYDV transmission efficiency is influenced by genetic diversity within the vector, identifying 542 single nucleotide polymorphisms that potentially contribute towards variable transmission efficiency in *Rh. padi*. Our results represent an important advancement in our understanding of the relationship between genetic diversity, vector–virus interactions, and virus transmission efficiency.

## 1. Introduction

Cereal aphids, including the grain aphid, *Sitobion avenae*, and the bird cherry-oat aphid, *Rhopalosiphum padi,* are important herbivorous insects of cereal crops [1]. Cereal aphids are widely distributed across central Europe and cause significant crop damage through feeding [2] and the transmission of plant viruses [3]. Cereal aphids vector several plant viruses, including those that cause yellow dwarf disease [4]. Yellow dwarf disease is caused by multiple viruses, including barley yellow dwarf virus (BYDV, Tombusviridae: *Luteovirus*), cereal yellow dwarf virus (CYDV, Solemoviridae: *Polerovirus*), maize yellow dwarf virus (MYDV, Solemoviridae: *Polerovirus*) and wheat yellow dwarf virus (WYDV, Solemoviridae) [5]. There are several virus species within each yellow dwarf virus genus [4,5]; however, the most agriculturally important in the UK and Europe is BYDV-PAV (Tombusviridae: *Luteovirus pavhordei*) [6]. Infection with BYDV-PAV can decrease crop yield by 20% [3,7]. Yellow dwarf disease symptoms include crop stunting, delayed crop maturity, shrivelled grain, reduced transpiration, and chlorosis [5].

Aphid and disease management strategies for yellow dwarf disease follow strict thresholds [8]. In the UK, the current threshold, the level of aphid infestation above which treatment is recommended, is the presence of a single virus-vectoring aphid in the crop during the early stages of plant growth [8,9]. Once the crop reaches growth stage 31, it is able to naturally tolerate yellow dwarf virus infection [10]. Similar stringent thresholds are followed in other European countries. These low thresholds probably contribute to increased application of management interventions such as insecticide treatments, directly increasing the development of insecticide resistant, or desensitized, aphid populations [11–13]. Currently, the same yellow dwarf virus threshold applies to all vector species, and all populations within a vector species. This is an important oversight, as aphid populations are not homogenous and there is inherent diversity within vector populations that can significantly influence the behaviour and phenology of both the aphid and the virus. Indeed, the transmission efficiency of BYDV-PAV differs between cereal aphid species, and a recent review found that transmission efficiency can also vary between aphid populations within a given species [5]. For example, transmission efficiency of BYDV-PAV by *Rh. padi* can range from 39% to 80%, 0% to 100% and 20% to 100% for wheat, barley and oats, respectively [5]. The biological drivers behind this variation are poorly understood; however, intra-species diversity (genetic diversity and the presence and diversity of endosymbionts) within aphid populations are some proposed hypotheses [5].

The majority of aphid species form an obligatory relationship with the endosymbiont *Buchnera aphidicola*. In this relationship, *B. aphidicola* supplements the diet of the host aphid through provision of amino acids [14]. Diversity within *B. aphidicola* can also influence other aspects of aphid fitness, conferring additional beneficial traits to the aphid such as heat tolerance [15]. Aphids can also form a range of non-essential, or facultative, relationships with several endosymbionts [16,17] that also influence aphid phenotype [18]. The facultative endosymbionts described to associate with aphids include *Regiella insecticola, Hamiltonella defensa, Fukatsuia symbiotica* (previously PAXS), *Serratia symbiotica, Rickettsia* spp., *Ricketsiella* spp., *Spiroplasma* spp. and *Arsenophonus* spp. [11,16,19,20]. Facultative endosymbionts occur naturally in cereal aphid populations [11,20], and facultative endosymbionts can exist in individual infections, co-infections or multi-infections [11,20–22]. Several fitness and behavioural traits can be conferred to the host aphid by facultative endosymbionts, including protection against parasitism [21] and differential feeding behaviour [23]. Endosymbiont effects can also be mediated by aphid genotype, through an endosymbiont–genotype interaction [22], and aphid genotype inherently influences aphid fitness [21].

**Figure 1.**
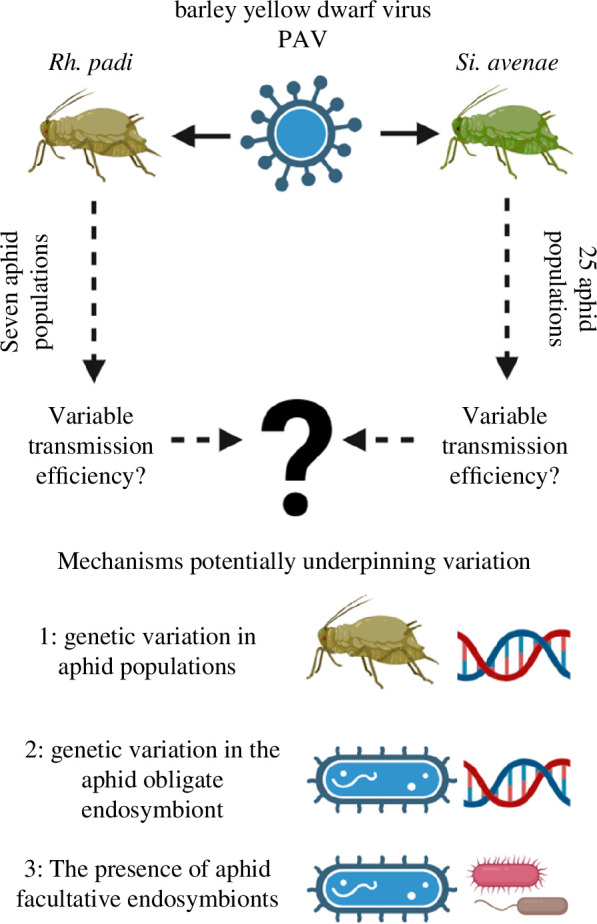
Graphical representation of the study system. Image created in bioRender (biorender.com).

Despite the broad effects intra-species diversity (genotype, *B. aphidicola* strain, facultative endosymbiont presence and strain) has on aphid phenology and behaviour, relatively few studies have examined how these traits impact aphid–virus interactions. Recent studies have started to explore the potential influence facultative endosymbionts might have on the aphid–BYDV relationship [24–26]; however, transmission efficiency is often not directly examined [25] or the observed endosymbiont effects cannot be disentangled from the confounding effect of aphid genotype [24]. Genetic variation has been found to underpin transmission efficiency in another aphid–yellow dwarf virus combination [27], but this remains understudied for *Rh. padi, Si. avenae* and BYDV-PAV. Here, we use Illumina sequencing to characterize genetic and endosymbiont diversity in aphid populations and combine this with BYDV transmission experiments to examine how diversity in vector populations impacts BYDV transmission efficiency. To achieve this, we use the most prevalent BYDV strain found in mainland Europe and the UK (BYDV-PAV) and several populations of the two most important vector species, *Rh. padi* (seven populations) and *Si. avenae* (25 populations). Figure 1 represents our study system. Broadly, our results provide biological insights into the drivers behind variable transmission efficiency in an important vector–virus system.

## 2. Methods

### 2.1. Characterization of intra-species diversity

Aphid populations comprised 25 Si. avenae populations and seven *Rh. padi* populations, an additional *Rh. padi* population (RP-12) was included in the genotyping analysis. We included this population to increase the genetic data available for our phylogenetic analyses. All aphid populations were maintained in cup cultures (similar to Leybourne *et al*. [21]) under controlled environmental conditions (18 ± 2°C; 16 L : 8 D cycle) in a plant growth room on *Triticum aestivum* cv. Alcedo. The sampling location for all aphid populations, along with their characterized facultative endosymbiont communities, was described previously [11].

For aphid genotyping, approximately 40 aphids (mixed life-stage) were collected into 96% molecular biology grade ethanol. Samples were sent to LGC Genomics GmbH (Berlin, Germany) for DNA extraction and sequencing (150 bp paired-end reads on an Illumina NextSeq 500/550 platform). All DNA extraction, library preparation and sequencing were conducted by LGC Genomics GmbH. Data processing and single nucleotide polymorphism (SNP) characterization were carried out at the Centre for Genomics Research (University of Liverpool).

### 2.2. Single nucleotide polymorphism calling

Aphid genomes were obtained from online databases, detailed in table 1. Aphid genomes were assessed for the presence of any symbiont genomes using blastn (using megablast algorithm [30], -evalue 1e−25) (v. 2.12.0+). *Buchnera aphidicola* contigs were identified and removed from the *Rh. padi* assembly.

**Table 1.** Access links and NCBI accessions for aphid and symbiont genomes.

organism	source (weblink or NCBI accession)
*Si. avenae*	https://figshare.com/collections/Grain_aphid_Sitobion_avenae_genomics/5425896/1 [28]
*B. aphidicola (Si. avenae*)	GCF_005082585.1
*Rh. padi*	https://bipaa.genouest.org/sp/rhopalosiphum_padi) [29]
*B. aphidicola (Rh. padi*)	GCF_005080845.1
*F. symbotica*	GCF_003122425.1
*Re. insecticola*	GCF_013373955.1
*H. defensa*	GCF_000021705.1

Reads were mapped with BWA MEM [31] (v. 0.7.17-r1188), and duplicate reads were marked with picard mark duplicates (v. 2.8.2). Variant calling and initial filtering were performed with samtools [32] (v. 1.6), bcftools [32] (v. 1.9), vcftools [33] (v.0.1.16) and snpEFF [34] (v. 5). Variants were initially filtered with a minor allele frequency of 0.05 to remove low-quality variants that are rare within the population, as well as a low cut-off of depth 2. For phylogenetic tree inference, variants were retained, where a genotype was called for each variant site in all samples. VCFs were thinned using vcftools to 1000 or 5000 bp for symbiont or aphid genomes, respectively. Any resulting SNPs were retained for multi-dimensional scaling (MDS) plot analysis in plink [35] (v. 1.9) and Newick tree generation using the python package VCF kit [36] (v. 0.2.9).

We used plink to perform linear regression and assess for SNPs associated with BYDV transmission and BYDV titre. For aphid samples, instead of using thinned VCFs, variants were thinned using ‘--indep 50 5 2’ to account for linkage disequilibrium (LD). LD pruning was not performed for symbiont samples. Phenotype association studies were performed in plink using the ‘--allow-no-sex --noweb --linear --ci 0.95’ options. Variants with a *p*-value of less than 0.05 were deemed to be of interest.

Phylogenetic distance between aphid and symbiont populations along the Newick tree and separation into distinct MDS clusters were used to assign our aphid populations into the putative aphid genotype, *B. aphidicola* and any associated secondary endosymbionts into putative microbial strains.

### 2.3. Barley yellow dwarf virus-PAV transmission experiments

Apterous adult aphids were randomly selected and placed onto BYDV-PAV-infected *T. aestivum* cv. Alcedo plants and left to feed for 48 h; source plants had a mean relative virus titre of 2.97 ± 0.62 and the BYDV-PAV culture held at the Julius Kühn Institut was used as a virus source [37]. Following this acquisition period, five adult apterous aphids from each population were selected and placed at the base of a new wheat plant (the BYDV-susceptible cv. Alcedo at Biologische Bundesanstalt, Bundessortenamt und Chemical Industry, growth stage 12 (BBCH)) for 48 h; virus-carrying aphids from the BYDV-PAV stock culture were used as a control. After 48 h, aphids were removed and plants were treated with insecticide. Plants were retained in the controlled environment chamber for six weeks for virus incubation. The plants were then screened for BYDV symptoms [5] and material was collected for serological detection of BYDV infection via a double antibody sandwich enzyme-linked immunosorbent assay (DAS-ELISA). The number of replicates per aphid population ranged from 21 to 24. Experiments were carried out in a controlled environment room (20 ± 2°C; 14 L : 10 D cycle). We used the highly efficient *Rh. padi* clone R07 [37] as a positive control in our transmission experiments.

For DAS-ELISA, 96 well polystyrene immunoassay microtiter plates were prepared by coating the plates with BYDV-specific polyclonal antibodies (IgG). BYDV-PAV IgG was prepared by the Julius Kühn Institut. The IgG concentration used was 1 : 200, diluted in ELISA coating buffer comprising: Na_2_CO_3_ (1.59 g l^-1^), NaHCO_3_ (2.93 g l^-1^), NaN_3_ (0.2 g l^-1^); pH 9.6. One hundred microlitres of IgG solution was added to each well, leaving two wells for blanks. Plates were incubated at 37°C for 4 h in a moist chamber. After incubation, plates were washed four times with wash buffer (Phosphate-buffered saline, (PBS): 40 g NaCl, 7.2 g Na_2_HPO_4_·2H_2_O, 1.0 g KH_2_PO_4_, 1.0 g KCl in 5 l, with 2.5 ml Tween; pH 7.3) using a plate washer (Tecan Hydrospeed, Crailsheim, Germany). Fifty milligrams of leaf tissue was sampled from each plant and placed in a 2 ml bead milling tube containing five steel beads. Samples were homogenized in 500 µl of extraction buffer (wash buffer + 2% polyvinylpyrrolidone K25 and 0.2% dry milk) through shaking in a Precellys^®^ Evolution homogenizer for 30 s at 25 000 r.p.m. A 100 µl aliquot of homogenate was placed in an IgG-coated well, and three negative controls (uninfected plant tissue) and three positive controls were included in each plate. Plates were covered and incubated at 4–6°C overnight. Plates were washed five times in wash buffer and the enzyme conjugate solution was added. The enzyme (alkaline phosphatase) conjugate solution comprised a 1 : 10 000 dilution of enzyme in extraction buffer. One hundred microlitres of enzyme conjugate solution was added to each well, and the plate was incubated at 37°C for 4 h. Plates were washed four times, and 200 µl of substrate buffer was added to each well. Substrate buffer comprised: 97 ml diethanolamine, 200 mg NaN_3_ and 203 mg MgCL_2_·6H_2_O in 1 l H_2_O + 1 mg ml^-1^ *p*-nitrophenyl phosphate; pH 9.8. Plates were incubated in the dark for 60 min; endpoint extinction was measured at 405 nm using a plate reader (Tecan Sunrise). Two blank wells (substrate buffer only) were included per plate, and all wells were blank corrected. The extinction intensity is a measure of the relative virus content/titre. The threshold for a positive BYDV-PAV infection was calculated at EXT of more than 0.06 (×(mean negative control) + 3 standard deviations (STD)).

### 2.4. Statistical analysis

All statistical analysis was carried out using R (v. 4.3.0) [38] and R Studio (v. 1.3.1093). The following additional packages were used to support data analysis and data visualization: car, v. 3.0-11 [39], ggplot2, v. 3.3.5 [40]. In all models, response variables included aphid genotype, *B. aphidicola* strain and facultative endosymbiont presence; *B. aphidicola* strain and facultative endosymbiont presence were tested as nested variables within aphid genotype. In the *Si. avenae* models, *Re. insecticola* strain was included as an additional explanatory variable (nested within aphid genotype).

BYDV transmission efficiency was analysed using a general linear model fitted with a binomial distribution and a logit link. A binary value (1 = infected; 0 = uninfected) was modelled as the response variable, and each aphid species was tested in a separate model. A Type II Wald *χ* ^2^ analysis of deviance test was used to test the model. Differences in viral inoculation (ELISA titre) were examined using linear models. The BYDV titre of successfully infected plants was modelled as the response variable, and each aphid species was tested in a separate model. A type II ANOVA was used to test the model.

## 3. Results

### 3.1. Genetic variation in *Rhopalosiphum padi* influences barley yellow dwarf virus transmission efficiency

**Figure 2.**
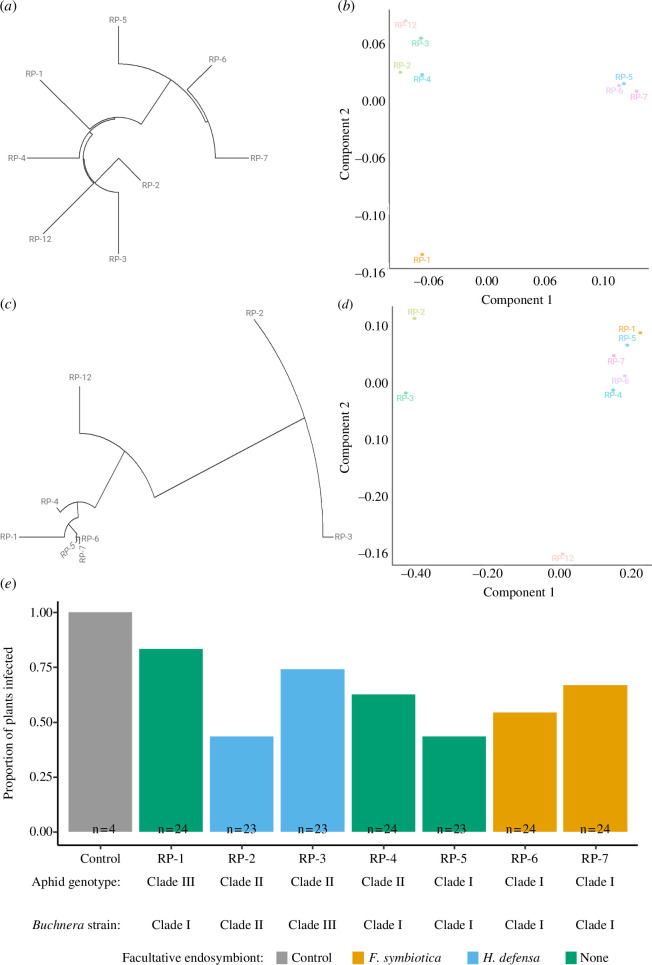
Newick tree (*a*, *b*) and multidimensional scaling (MDS) clustering (*b*, *d*) for *Rh. padi* (*a*, *b*) and *B. aphidicola* (*c*, *d*) based on SNPs. (*e*) The transmission efficiency for each *Rh. padi* population and the internal control; colour shows facultative endosymbiont presence, and *n* represents the number of replicates.

From the Illumina data, we identified 6444 *Rh*. *padi* SNPs and used these to group the *Rh. padi* populations into three genetically similar clades (figure 2*a,b*). We used these clusters to call putative genotypes (clades) for the *Rh. padi* populations. We also observed clustering for the obligatory endosymbiont *B. aphidicola* (figure 2*c,d*) and called putative strains for these based on 34 SNPs.

We detected significant variation in BYDV transmission efficiency between the *Rh. padi* populations examined (figure 2*e*), with differences attributed to aphid genotype ( $\chi_{2}^{2}$ = 6.12; *p* = 0.046). On average, aphids in clade I were the least efficient BYDV-PAV vectors and clade III the most efficient vector. We identified 542 SNPs that could potentially contribute to variable transmission efficiency in *Rh. padi. Buchnera aphidicola* ( $\chi_{1}^{2}$ = 5.53; *p* = 0.063) and facultative endosymbiont presence ( $\chi_{1}^{2}$ = 2.38; *p* = 0.123) had no observable effect on BYDV transmission efficiency in *Rh. padi*. We did not detect any effect of aphid genotype (*F*_2.95_ = 1.92; *p* = 0.151), *B. aphidicola* strain (*F*_2.95_ = 0.61; *p* = 0.543) or facultative endosymbiont presence (*F*_1.95_ = 0.90; *p* = 0.345) on BYDV titre inoculated into the plant tissue following successful transmission (electronic supplementary material, figure S1*a*).

### 3.2. Transmission of barley yellow dwarf virus-PAV by *Sitobion avenae* is broadly inefficient and not affected by vector diversity

**Figure 3.**
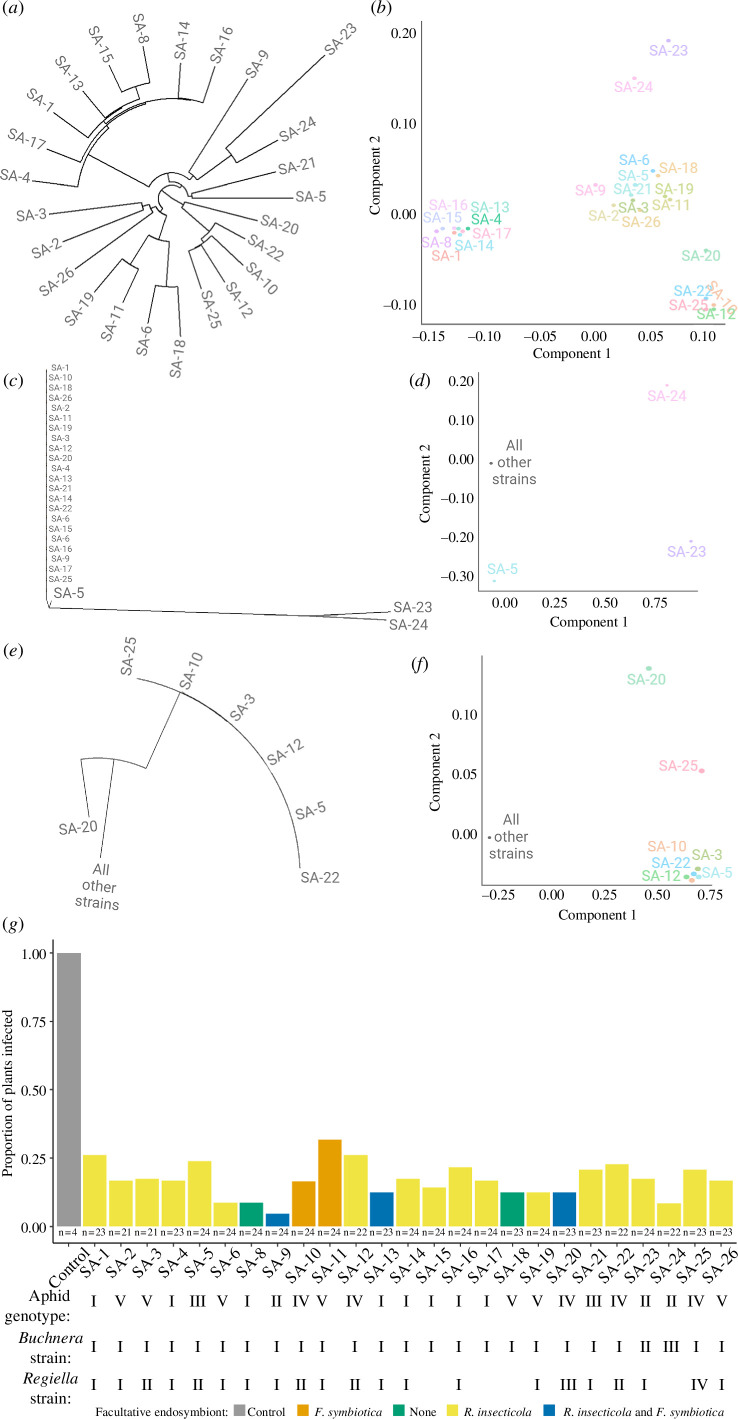
Newick tree (*a*, *c*, *e*) and multidimensional scaling (MDS) clustering (*b*, *d*, *f*) for *Si. avenae* (*a*, *b*), *B. aphidicola* (*c*, *d*), and *Re. insecticola* (*e*, *f*) based on SNPs. (*g*) The transmission efficiency for each *Si. avenae* population and the internal control; colour shows facultative endosymbiont presence, and *n* represents the number of replicates.

We identified 5274 SNPs in our *Si. avenae* Illumina data, and the 25 *Si. avenae* populations clustered into several clades (figure 3*a*,*b*). In contrast with *Rh. padi*, we did not observe a high level of genetic diversity across the obligatory endosymbiont *B. aphidicola,* with the majority of *B. aphidicola* strains grouping together based on eight SNPs (figure 3*c,d*). However, we detected genetic variation in the facultative endosymbiont, *Re. insecticola,* with four putative strains based on 29 SNPs (figure 3*e,f*).

For the *Si. avenae* populations examined (figure 2*g*), we observed no effect of aphid genotype ( $\chi_{4}^{2}$ = 5.71; *p* = 0.222), *B. aphidicola* strain ( $\chi_{2}^{2}$ = 0.84; *p* = 0.656), facultative endosymbiont presence ( $\chi_{5}^{2}$ = 1.74; *p* = 0.884) or *Re. insecticola* strain ( $\chi_{4}^{2}$ = 1.76; *p* = 0.778) on BYDV-PAV transmission efficiency. When compared with *Rh. padi* (figure 2*e*), all *Si. avenae* clones examined (figure 3*g*) were more inefficient at vectoring BYDV-PAV. We did not detect any effect of aphid genotype (*F*_4.82_ = 0.85; *p* = 0.495), *B. aphidicola* strain (*F*_2,82_ = 0.30; *p* = 0.739), facultative endosymbiont presence (*F*_5,82_ = 0.86; *p* = 0.510) or *Re. insecticola* strain (*F*_4.82_ = 0.67; *p* = 0.609) on BYDV titre inoculated into the plant tissue following a successful transmission (electronic supplementary material, figure S1*b*).

## 4. Discussion

Our work presents an investigation into the influence that diversity in vector populations has on the transmission efficiency of an important cereal virus. We find that the two vector species examined, *Rh. padi* and *Si. avenae*, can be broadly categorized into highly efficient and moderately efficient vectors of BYDV-PAV, respectively. For the efficient vector, *Rh. padi*, we show that virus transmission efficiency is influenced by genetic variation, and we identify 542 SNPs that potentially influence virus transmission. Broadly, our findings help disentangle the relationship between vector diversity and the transmission efficiency of important plant viruses and provide insights that can guide future research endeavours.

A recent review synthesized information on the transmission efficiency of yellow dwarf virus across the main cereal aphid vectors, including *Rh. padi* and *Si. avenae* [5], identifying significant variation in transmission efficiency across virus species and strains, vector species and different clonal populations within a vector species [5]. Variation in transmission efficiency in different vector–virus and virus–host (plant) combinations is unsurprising, as vector–virus relationships can be highly specific. Vectors can be characterized as efficient (competent) or inefficient (incompetent) vectors for a given virus strain. For our study, we selected *Rh. padi* and *Si. avenae* as these species are considered to be important and efficient vectors for BYDV-PAV [5,41,42]. However, as we observed consistently low levels of transmission efficiency of BYDV-PAV by *Si. avenae*, this aphid species might only be a moderately efficient vector for BYDV-PAV when compared with *Rh. padi*. It should be noted that although we observed low transmission efficiency of BYDV-PAV in *Si. avenae* and high efficiency for *Rh. padi*, this might differ for other BYDV species. For example, *Si. avenae* is known to be an efficient vector of BYDV-MAV (Tombusviridae: *Luteovirus mavhordei*) [5]; therefore, while we observe low BYDV-PAV efficiency in the *Si. avenae* lines tested here, transmission efficiency might be more variable for other yellow dwarf virus species.

For *Rh. padi,* we observed significant variation in BYDV-PAV transmission efficiency between the populations examined, with transmission efficiency ranging from 40% to 80%. This broadly supports previous observations that transmission efficiency varies significantly between clonal populations within a cereal aphid species [5,37,43]. Three mechanisms that could potentially explain this variation were recently proposed [5]. These included: (i) the indirect effect of facultative endosymbionts through altered aphid feeding and probing behaviour; (ii) the direct effect of *B. aphidicola* through variation in endosymbiont-derived chaperonin proteins; and (iii) aphid genetic variation and the presence of vectoring alleles. Our study represents an examination of these hypotheses in *Rh. padi* and *Si. avenae* and identifies genetic diversity as a key factor underpinning transmission efficiency in *Rh. padi*. We excluded behavioural plasticity as a source for varying BYDV-PAV transmission efficiency, as it is low for feeding behaviour associated with virus acquisition and transmission in *Si. avenae* [44].

Our observation of differential BYDV-PAV transmission efficiency across our *Rh. padi* genotypes complements results reported for the wheat aphid, *Schizaphis graminum* [27,45–47]. Previous research used efficient and inefficient *Sc. graminum* populations and two other yellow dwarf virus species, CYDV-RPV (Solemoviridae: Polerovirus) and WYDV-SGV (Solemoviridae) to examine how genetic traits influence virus transmission efficiency [27,45–48]. This process identified ‘vectoring alleles’ that underpin efficient CYDV-RPV transmission in *Sc. graminum* [27] and provides evidence that genetic diversity within vector populations is a key driver of transmission efficiency [45–48], as found here for our *Rh. padi* populations. We identified 542 SNPs in *Rh. padi* that are probably involved in underpinning variation in BYDV-PAV transmission efficiency. However, it should be noted that our observations are based on a relatively small number of aphid populations and that information on additional populations is required in order to fully elucidate the genetic traits underpinning transmission efficiency in *Rh. padi*. Nonetheless, to the best of our knowledge, no other studies have characterized genetic diversity within different vector populations and linked this with variation in yellow dwarf virus transmission efficiency in *Rh. padi* [5]. Therefore, the previous insights gained in the *Sc. graminum* (CYDV-RPV) and *Sc. graminum* (BYDV-SGV) systems, and our new observations in the *Rh. padi* (BYDV-PAV) system, represent important advancements in our understanding of the relationship between genetic diversity, vector–virus interactions and transmission efficiency.

We found no evidence to support the two other hypotheses recently proposed [5]: (i) indirect effect of facultative endosymbionts through altered aphid feeding and probing behaviour; and (ii) direct effect of *B. aphidicola* through variation in endosymbiont-derived chaperonin proteins. However, some of the phenotypic traits conferred by facultative endosymbionts can act in a synergistic manner with host aphid genotype [22,49]. Therefore, future work that explores endosymbiont effects on BYDV transmission in interaction with aphid genotype, while controlling for host aphid genotype, would enable a more robust examination of these hypotheses. For example, future work could disentangle potential interactive effects by investigating the potential aphid genotype × endosymbiont effects by manipulating the endosymbionts of aphids from clade III (high efficiency) and clade I (low efficiency) through elimination (antimicrobial treatment) and introduction (microinjection) of different *B. aphidicola* strains and facultative endosymbiont species.

## Data Availability

Metadata, transmission data and R analysis code are available via the University of Liverpool Data Catalogue [50]. Illumina data have been deposited to the European Nucleotide Archive (ENA) database, project ID PRJEB72361. Informatics code used in the project are available via Zenodo [51]. Electronic supplementary material is available online [52].
